# Diabetes-related ten-year outcomes after percutaneous coronary intervention of in-stent restenosis

**DOI:** 10.1007/s00392-025-02782-6

**Published:** 2025-11-03

**Authors:** Constantin Kuna, Eduard Braun, Christian Bradaric, Tobias Koch, Antonia Presch, Felix Voll, Sebastian Kufner, Tareq Ibrahim, Heribert Schunkert, Karl-Ludwig Laugwitz, Salvatore Cassese, Adnan Kastrati, Jens Wiebe

**Affiliations:** 1 Department of Cardiology, Deutsches Herzzentrum München, Universitätsklinikum Der Technischen Universität München, Munich, Germany; 2 DZHK (German Centre for Cardiovascular Research), Partner Site Munich Heart Alliance, Munich, Germany; 3 Clinic and Policlinic Internal Medicine I (Cardiology and Angiology), Universitätsklinikum Der Technischen Universität München, Klinikum Rechts Der Isar, Munich, Germany; 4 Helios Klinikum München West, Munich, Germany

**Keywords:** Diabetes mellitus, Drug-coated balloon, Drug-eluting stent, In-stent restenosis, Percutaneous coronary intervention

## Abstract

**Background:**

Limited data is available for long-term outcomes after percutaneous coronary intervention (PCI) of coronary drug-eluting stent (DES) in-stent restenosis (ISR) in diabetics.

**Aims:**

Thus, the aim of this observational, retrospective study was to close this lack of evidence.

**Methods:**

Between January 2007 and February 2021, a total of 3511 patients with 5497 ISR lesions were treated at two large-volume centers in Munich, Germany, of which 1242 (35.4%) were diabetics. Endpoints of interest were the rates of cardiac death, repeat revascularization, and myocardial infarction (MI). Survival was analyzed using the Kaplan–Meier method. Differences between the groups were tested with the log-rank test. Conventional multivariable analysis with adjustment for relevant variables was performed.

**Results:**

After 10 years, the rates of cardiac death were 42.8% for diabetics and 32.8% for nondiabetics (HR_adj_ 1.55 [95% CI, 1.31–1.81], *p* < 0.001). MI occurred in 15.9% of diabetics and in 9.7% of non-diabetics (HR_adj_ 1.70 [95% CI, 1.36–2.11], *p* < 0.001). The rates of repeat revascularization of target lesion (HR_adj_ 1.17 [95% CI, 1.02–1.34], *p* = 0.028), target vessel, and nontarget vessel were significantly higher in diabetics as compared to nondiabetics. No statistically relevant difference was found regarding the rate of stent thrombosis. Compared to non-insulin-dependent diabetics, insulin-dependent diabetics show higher rates of cardiac death and MI, but comparable revascularization rates in both diabetic groups.

**Conclusions:**

In the long term, the rates of cardiac death, MI, and repeat revascularization after PCI of DES-ISR are significantly higher in diabetics, particularly in insulin-dependent diabetics, than in nondiabetics.

**Graphical Abstract:**

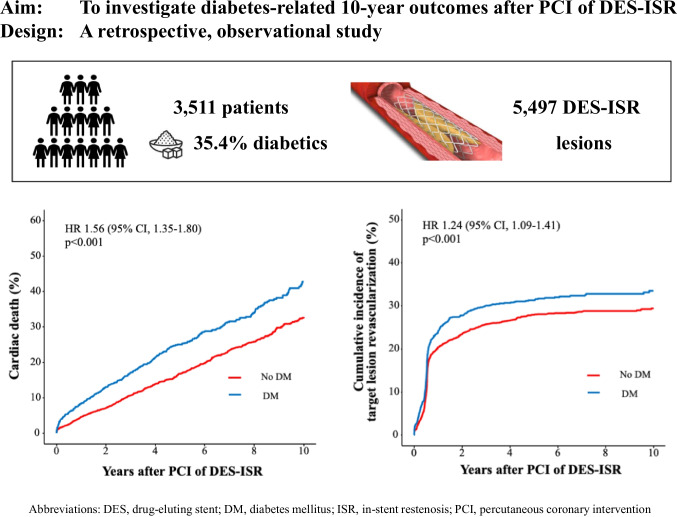

**Supplementary Information:**

The online version contains supplementary material available at 10.1007/s00392-025-02782-6.

## Introduction

Globally, coronary artery disease (CAD) is a leading cause of death, with percutaneous coronary intervention (PCI) as an established revascularization procedure [1]. The occurrence of in-stent restenosis (ISR) and the need for repeat revascularization are quantified as 1–2% per year, limiting long-term outcomes after PCI [2]. The implementation of newer generation drug-eluting stents (DES) has substantially reduced the need for target lesion revascularization (TLR); however, ISR remains an important issue in clinical practice, being associated with elevated mortality rates [3]. About 10% of all PCI cases are attributable to the intervention of ISR[4]; repeat DES implantation and drug-coated balloon (DCB) angioplasty represent the most effective treatment options for DES-ISR [5]. Compared to PCI for native coronary stenosis, PCI of ISR is accompanied by increased rates of adverse events [6].

Based on data from 2021, 537 million people worldwide suffer from diabetes mellitus (DM), predicted to rise to 783 million in 2045, illustrating the importance of this cardiovascular risk factor [7]. Hyperglycemia and insulin resistance have been shown to strongly predict the occurrence of macrovascular disease and to increase the risk of CAD [8]. DM is considered a predictor for poor clinical outcomes following PCI in coronary de novo lesions, going along with elevated prevalence of cardiac death, myocardial infarction (MI), and repeat revascularization [9, 10]. The correlation between DM and ISR still is a matter of debate. While some trials did not confirm a link between DM and the occurrence of DES-ISR [11, 12], others revealed DM to be accompanied by increased ISR rates and to predict recurrent ISR [4, 13, 14]. Diabetics with DES-ISR face “double jeopardy” as they are subjected to the risk of DM as a strong cardiovascular risk factor on the one hand, and exposed to ISR, a condition clearly associated with worse outcomes on the other hand.

Due to the steadily increasing prevalence of DM and the strong correlation with CAD, there is great interest to further illuminate the impact of DM on clinical long-term outcomes after PCI of ISR as the clinical relevance could be even higher in the future [15]. As most outcome data after PCI for patients with DM refer to the treatment of native coronary artery stenosis, data analyzing clinical long-term outcomes after PCI of DES-ISR in diabetics are scarce and mostly limited in patient numbers and in follow-up [14, 16, 17]. Thus, the aim of this analysis is to compare outcomes after PCI of DES-ISR between a large cohort of diabetics and nondiabetics in the long term.

## Methods

### Study design and patient selection

The ISAR-DESIRE registry is an observational, retrospective study of patients who underwent PCI for the treatment of ISR between January 2007 and February 2021 at the Deutsches Herzzentrum München, Munich, Germany and the 1. Med. Klinik, Klinikum rechts der Isar, Technical University, Munich, Germany. The present analysis focuses on the impact of DM on long-term outcomes after PCI of DES-ISR. DM was diagnosed based on the American Diabetes Association definitions in patients who were on treatment with insulin or oral antidiabetic drugs, who had a fasting blood glucose level of ≥ 126 mg/dl, an abnormal blood glucose level of ≥ 200 mg/dl documented 2 h after a glucose tolerance test, or an HbA1c of ≥ 6.5% [18]. The study was approved by the local ethics board of the Technical University Munich, Germany, and adheres to the Declaration of Helsinki.

### Interventional procedure and medications

PCI of DES-ISR was performed according to standard clinical practice. All patients were treated with either balloon angioplasty (BA) (including plain old balloon angioplasty and drug-coated balloon (DCB) angioplasty) or implantation of DES. The decision regarding the individual therapy strategy was determined by the treating physician. According to local standards, predilatation was recommended for all patients undergoing PCI for ISR; the use of scoring or debulking devices was left to the operators’ discretion, as well as the decision to perform post-dilatation after stenting. During the procedure, patients usually received 500 mg aspirin intravenously and body weight adjusted intra-arterial or intravenous heparin or bivalirudin immediately after the decision to perform the intervention. After the intervention, all patients received aspirin indefinitely and a P2Y12 inhibitor (clopidogrel, prasugrel, or ticagrelor) according to clinical presentation and recommendations at the time of PCI.

### Data collection

Personnel of the clinical data management center (ISAResearch-Center, Munich, Germany) collected relevant data on source-documented hospital chart reviews. This data included information regarding clinical and lesion-related parameters at the time of initial and repetitive PCIs. The required data for this study was entered into an anonymized study database. Angiographic parameters of interest included the type of intervention (BA vs. DES), balloon diameter and maximum balloon pressure, total stented length, and stent diameter, as well as thrombolysis in myocardial infarction (TIMI) grade flow pre- and post-PCI.

### Endpoints

Endpoints of interest regarding this diabetes-related analysis were the rates of all-cause death, cardiac death, myocardial infarction (MI), target lesion revascularization (TLR), target vessel revascularization (TVR), nontarget vessel revascularization (NTVR), and stent thrombosis (ST). TVR was defined as revascularization in the target vessel outside the target lesion. Event rates are presented as Kaplan–Meier 10-year event rates for all-cause death and cardiac death, and cumulative ten-year incidences after accounting for the competing risk of death for the other endpoints. The definition of myocardial infarction (MI) used in this analysis was based on the Third Universal Definition of Myocardial Infarction [19]. The definitions of ISR, ST, TLR, and NTVR used in this trial were adapted from the Academic Research Consortium [20, 21].

### Statistics

Baseline descriptive parameters are presented as mean ± standard deviation for continuous data and numbers with percentages for categorical data. The Cox proportional hazards model for time-to-first-event analysis was used to calculate hazard ratios and 95% confidence intervals. An extended Cox model (Andersen–Gill model) was used for recurrent TLR. All-cause and cardiac mortality rates were obtained by the Kaplan–Meier estimates. Incidences of all other events were calculated by accounting for the competing risk of death and for within-subject correlation by using robust sandwich estimators for the variance of regression coefficients. The treatment of the first DES-ISR was defined as time zero. Univariate analysis considering clinical and procedure-related variables was performed. Conventional multivariable analyses were performed with adjustment for the following variables: age, sex, multivessel CAD, prior MI, prior CABG, ejection fraction, use of DCB, creatinine and C-reactive protein on admission, acetylsalicylic acid, P2Y12 inhibitor, and statin on discharge. A 2-tailed *p* value of less than 0.05 was considered statistically significant. Statistical software R (Version 4.0, R Foundation for Statistical Computing, Vienna, Austria) was used for all analyses.

## Results

### Baseline and procedural characteristics

A total of 3511 patients underwent PCI due to ISR; 35.4% (1242 patients) suffered from DM. Diabetics were more often male than nondiabetics (76.2 vs. 79.4; *p* = 0.029), presented with a higher body mass index (28.6 kg/m^2^ vs. 26.9 kg/m^2^; *p* < 0.001), and more often suffered from arterial hypertension (97.9% vs. 90.4%; *p* < 0.001). Diabetics were more likely to suffer from coronary multivessel disease (93.4% vs. 87.6%; *p* < 0.001) and to present with a history of coronary artery bypass graft (CABG) operation (15.5% vs. 13.0%; *p* = 0.054). Diabetics and nondiabetics equally commonly presented with acute coronary syndrome (ACS) (30.2% vs. 28.8%; *p* = 0.416). Moreover, diabetics presented with a lower ejection fraction (51.4% vs. 52.8%; *p* < 0.001) and higher creatinine at admission (1.12 mg/dl vs. 1.11 mg/dl; *p* ≤ 0.010). Further clinical baseline characteristics are presented in Table 1. The division of clinical baseline characteristics in patients with insulin-dependent diabetes mellitus (IDDM), non-insulin-dependent diabetes mellitus (NIDDM), and nondiabetics is shown in Table S1.

**Table 1:** Clinical baseline characteristics

	Diabetics (*n* = 1242)	Non-diabetics (n = 2269)	*p* value
Age (in years)	69.6 ± 9.7	69.0 ± 10.9	0.075
Male sex	76.2 (946)	79.4 (1,802)	0.029
BMI (in kg/m^2^)	28.6 ± 4.8(*n* = 1233)	26.9 ± 4.2(*n* = 2198)	< 0.001
Arterial hypertension	97.9 (1216)	90.4 (2052)	< 0.001
Hypercholesterolemia	76.3 (948)	72.2 (1639)	0.009
Current smoker	15.3 (190)	14.5 (330)	0.581
CAD			< 0.001
- 1-vessel CAD - 2-vessel CAD - 3-vessel CAD	6.6 (82) 17.6 (219) 75.8 (941)	12.4 (282) 21.5 (488) 66.1 (1499)	
Multivessel CAD	93.4 (1160)	87.6 (1987)	< 0.001
Prior MI	43.6 (541)	45.9 (1042)	0.190
Prior CABG	15.5 (192)	13.0 (296)	0.054
Ejection fraction (in %)	51.4 ± 11.5	52.8 ± 10.9	< 0.001
ACS	30.2 (375)	28.8 (654)	0.416
Clinical presentation			< 0.001
- Stable angina - Unstable angina - NSTEMI - STEMI	69.8 (867) 12.0 (149) 15.7 (195) 2.5 (31)	71.2 (1615) 15.8 (358) 10.2 (232) 2.8 (64)	
Creatinine ad admission (in mg/dl)	1.12 ± 0.44	1.11 ± 0.39	0.010
CRP ad admission (in mg/l)	5.5 ± 17.0	4.9 ± 14.1	0.326
ASA at discharge	96.4 (1197)	97.0 (2200)	0.406
P2Y12 inhibitor at discharge	95.3 (1184)	94.6 (2147)	0.408
Oral anticoagulation at discharge	13.6 (169)	14.3 (324)	0.619
Statin at discharge	90.5 (1124)	91.8 (2082)	0.229

Data shown as percentage (number) or means ± standard deviation

*ACS*, acute coronary syndrome; *ASA*, acetylsalicylic acid; *BMI*, body mass index; *CABG*, coronary artery bypass graft; *CAD*, coronary artery disease; *CRP*, C-reactive protein; *MI*, myocardial infarction; *NSTEMI*, non-ST-elevation myocardial infarction; *STEMI*, ST-elevation myocardial infarction

A total of 5497 ISR lesions were treated. In diabetics, ISR was treated with DES implantation in 51.2% and in nondiabetics in 54.7% (*p* = 0.011). In the BA group, 44.3% (449/1013 patients) of diabetics and 46.4% (534/1550 patients) of nondiabetics were treated with a DCB. Mean maximum balloon diameter was 3.29 ± 0.61 mm in diabetics and 3.33 ± 0.61 mm in nondiabetics (*p* = 0.140). Mean maximum balloon pressure was 16.0 ± 4.2 atm in diabetics and 15.8 ± 4.1 atm in nondiabetics (*p* = 0.218). Mean maximum stent diameter was 3.24 ± 0.55 mm in diabetics and 3.28 ± 0.52 mm in nondiabetics (*p* = 0.174). Total stented length was 25.2 ± 13.6 mm in diabetics and 25.6 ± 13.2 mm in nondiabetics (*p* = 0.529). A summary of procedure-related characteristics is shown in Table 2. The division of procedure-related baseline characteristics in patients with IDDM, NIDDM, and nondiabetics is presented in Table S2. While the rate of DES implantation for the treatment of DES-ISR slightly decreased, the rate of DCB use strongly raised over the time of study inclusion (Figure S1).

**Table 2:** Procedural characteristics

	Lesions in diabetics (n = 2074)	Lesions in nondiabetics (*n* = 3423)	*p* value
Type of intervention			0.011
- Balloon angioplasty - DES implantation	48.8 (1013) 51.2 (1061)	45.3 (1550) 54.7 (1873)	
Drug-coated balloon	21.6 (449)	15.6 (534)	< 0.001
Cutting balloon	2.7 (55)	2.6 (88)	0.924
Bifurcation lesion	34.2 (708) (*n* = 2072)	32.5 (1105) (*n* = 3395)	0.228
Ostial lesion	32.0 (664) (*n* = 2073)	30.0 (1019) (*n* = 3398)	0.119
Max. balloon diameter (mm)	3.29 ± 0.61 (*n* = 2040)	3.33 ± 0.61 (*n* = 3305)	0.140
Max. balloon pressure (atm)	16.0 ± 4.2 (*n* = 2040)	15.8 ± 4.1 (*n* = 3284)	0.218
Max. stent diameter (mm)	3.24 ± 0.55 (*n* = 1072)	3.28 ± 0.52 (*n* = 1861)	0.174
Total stented length (mm)	25.2 ± 13.6 (*n* = 175)	25.6 ± 13.2 (*n* = 1861)	0.529
TIMI grade flow pre-PCI			0.156
- 0 - 1 - 2 - 3	6.8 (141) 1.9 (40) 7.3 (151) 84.0 (1741)	8.2 (280) 1.9 (66) 6.3 (215) 83.5 (2847)	
TIMI grade flow post-PCI			0.289
- 0 - 1 - 2 - 3	1.5 (31) 0.2 (4) 2.0 (41) 96.3 (1997)	2.0 (68) 0.4 (14) 2.0 (69) 95.6 (3254)	

Data shown as percentage (number) or means ± standard deviation

*DES*, drug-eluting stent; *PCI*, percutaneous coronary intervention; *TIMI*, thrombolysis in myocardial infarction

### Clinical outcomes

**Fig. 1:**
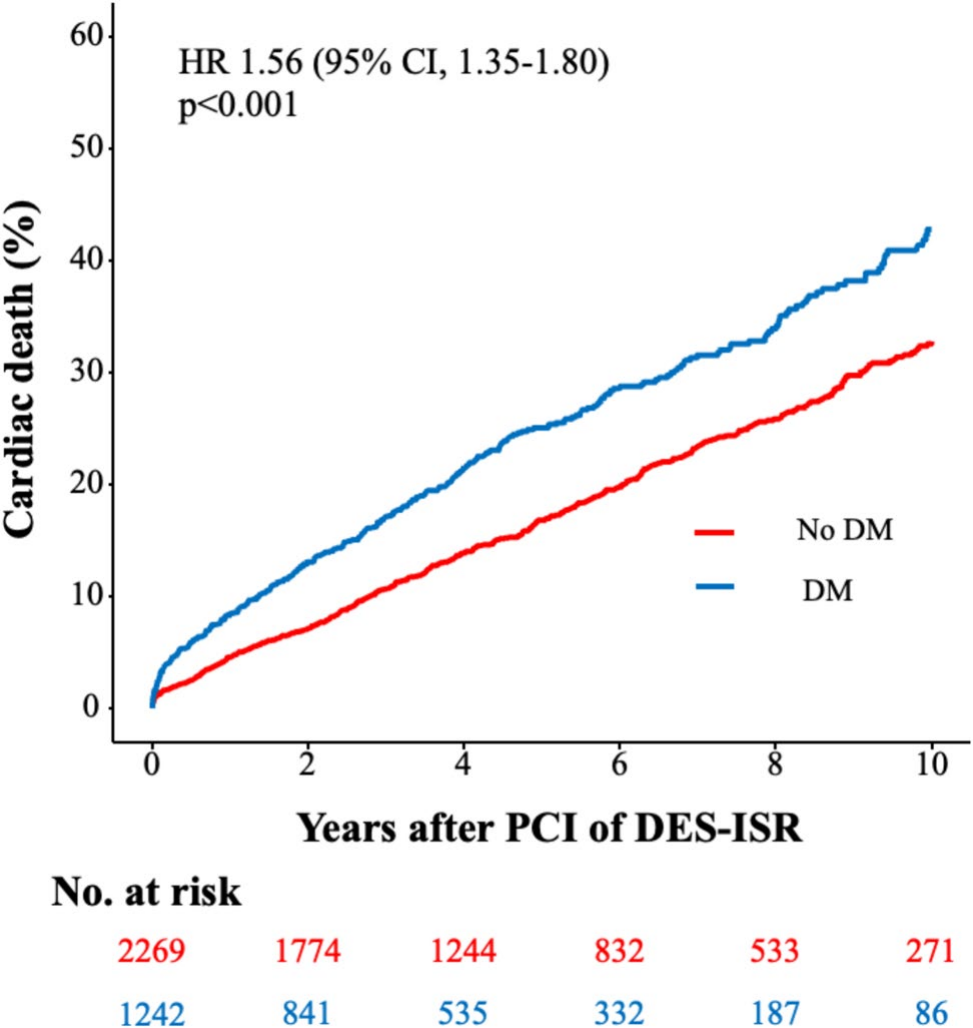
Diabetes-related ten-year incidence of cardiac death. The figure demonstrates the cumulative incidence function curve and HR with accompanying 95% CI

**Fig. 2:**
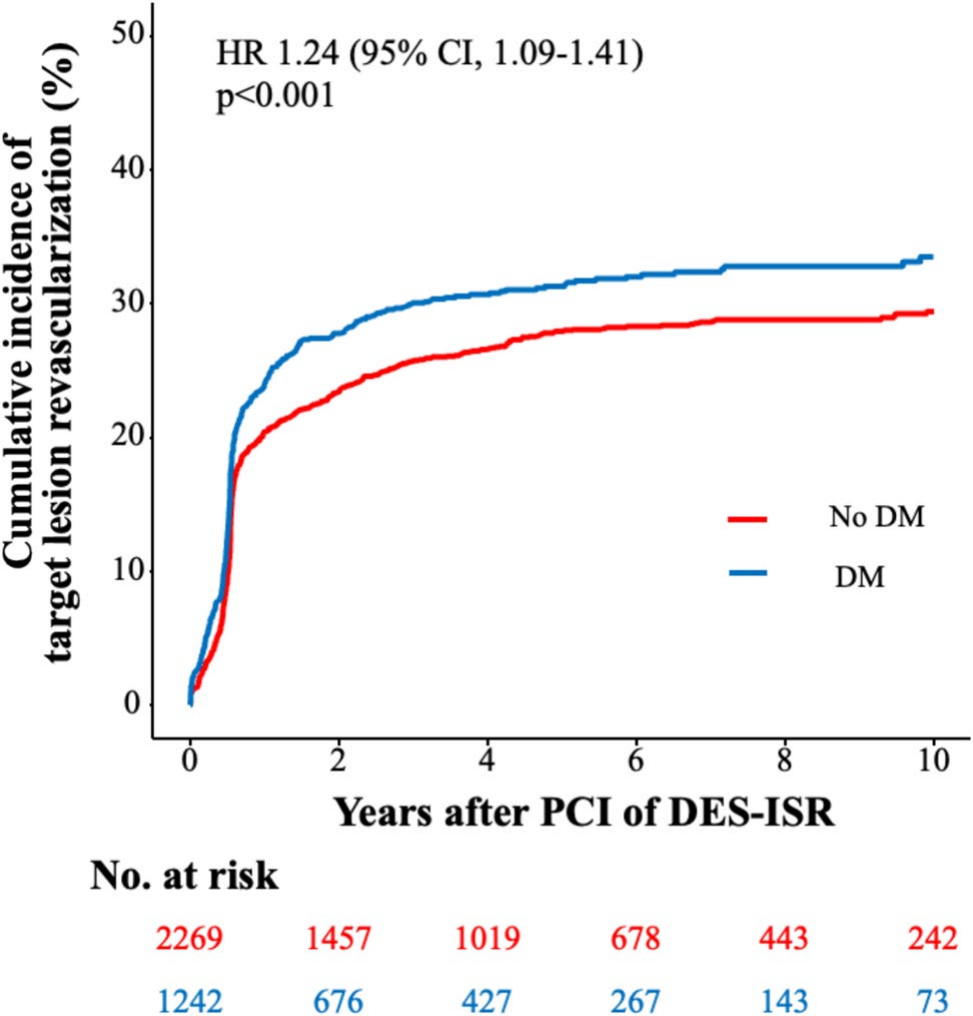
Diabetes-related ten-year cumulative incidence of TLR. The figures demonstrate the cumulative incidence function curves and HR with accompanying 95% CI

The median follow-up duration was 5.9 years (75% confidence interval (CI): 3.5; 9.4 years). Then, 19% of the total patient cohort (670 of 3511 patients) had a follow-up of 9 years or less. The rate of 10-year all-cause death after PCI of ISR was significantly higher in diabetics than in nondiabetics (57.7% vs. 43.4%; HR 1.59 [95% CI, 1.40–1.79], *p* < 0.001; adjusted HR (HR_adj_) 1.56 [95% CI, 1.38–1.77], *p* < 0.001). Moreover, the rate of 10-year cardiac death after PCI of ISR was significantly higher in diabetics than in nondiabetics (42.8% vs. 32.8%; HR 1.56 [95% CI, 1.35–1.80], *p* < 0.001; HR_adj_ 1.55 [95% CI, 1.31–1.81], *p* < 0.001) (Fig. 1). Significantly, more diabetics underwent TLR after 10 years than nondiabetics (33.5% vs. 29.4%; HR 1.24 [95% CI, 1.09–1.41], *p* < 0.001; HR_adj_ 1.17 [95% CI, 1.02–1.34], *p* = 0.028) (Fig. 2). MI rate was 15.9% in diabetics and 9.7% in nondiabetics (HR 1.78 [95% CI, 1.45–2.20], *p* < 0.001; HR_adj_ 1.70 [95% CI, 1.36–2.11], *p* < 0.001) (Figure S2). At 10-year follow-up, the TVR rate was 29.9% in diabetics and 25.2% in nondiabetics (HR 1.33 [95% CI, 1.16–1.52], *p* < 0.001; HR_adj_ 1.26 [95% CI, 1.09–1.46], *p* = 0.002). NTVR rate was 43.6% in diabetics and 36.4% in nondiabetics (HR 1.38 [95% CI, 1.23–1.55], *p* < 0.001; HR_adj_ 1.26 [95% CI, 1.12–1.42], *p* < 0.001). After 10 years, ST was detected in 2.3% of diabetics and in 1.6% of nondiabetics (HR 1.51 [95% CI, 0.92–2.49], *p* = 0.106; HR_adj_ 1.57 [95% CI, 0.95–2.59], *p* = 0.081). An overview of the clinical outcomes is presented in Table 3.

**Table 3:** Clinical outcomes

	PCI in diabetics (*n* = 1242)	PCI in nondiabetics (*n* = 2269)	HR (95% CI)	Unadjusted *p* value	HR_adj_ (95% CI)	Adjusted *p* value
All-cause death	442 (57.7)	622 (43.4)	1.59 (1.40–1.79)	< 0.001	1.56 (1.38–1.77)	< 0.001
Cardiac death	318 (42.8)	458 (32.8)	1.56 (1.35–1.80)	< 0.001	1.55 (1.33–1.81)	< 0.001
MI	164 (15.9)	191 (9.7)	1.78 (1.45–2.20)	< 0.001	1.70 (1.36–2.11)	< 0.001
TLR	375 (33.5)	613 (29.4)	1.24 (1.09–1.41)	< 0.001	1.17 (1.02–1.34)	0.028
TVR	330 (29.9)	515 (25.2)	1.33 (1.16–1.52)	< 0.001	1.26 (1.09–1.46)	0.002
NTVR	483 (43.6)	737 (36.4)	1.38 (1.23–1.55)	< 0.001	1.26 (1.12–1.42)	< 0.001
Definite ST	27 (2.3)	35 (1.6)	1.51 (0.92–2.49)	0.106	1.57 (0.95–2.59)	0.081

The numbers shown in brackets are Kaplan–Meier estimates (%). Cumulative incidence functions were computed for outcomes other than death to account for competing risks. The adjusted hazard ratios, 95% CI, and *P* values reported here are derived from a conventional multivariable analysis with adjustment for the following variables: age, sex, multivessel CAD, prior MI, prior CABG, ejection fraction, use of DCB, creatinine and C-reactive protein ad admission, acetylsalicylic acid, P2Y12 inhibitor and statin at discharge

Target vessel revascularization is defined as revascularization in the target vessel outside the target lesion

*CABG*, coronary artery bypass graft; *CAD*, coronary artery disease; *DCB*, drug-coated balloon; HR, hazard ratio; *HR*_adj_, adjusted hazard ratio; *MI*, myocardial infarction; *NTVR*, nontarget vessel revascularization; *PCI*, percutaneous coronary intervention; *TLR*, target lesion revascularization; *TVR*, target vessel revascularization; *ST*, stent thrombosis

By dividing the diabetic patient group into patients with IDDM and NIDDM, one can recognize that the comparison of either of the two diabetic groups to nondiabetics revealed significantly higher rates of cardiac death (HR 1.88 [95% CI, 1.55–2.28], *p* < 0.001; HR 1.39 [95% CI, 1.17–1.65], *p* < 0.001) and all-cause death (HR 1.91 [95% CI, 1.62–2.25], *p* < 0.001; HR 1.41 [95% CI, 1.22–1.63], *p* < 0.001) as well as of MI (HR 2.17 [95% CI, 1.65–2.84], *p* < 0.001; HR 1.57 [95% CI, 1.22–2.01], *p* < 0.001) and repeat revascularization in the diabetic group (TLR: HR 1.37 [95% CI, 1.14–1.64], *p* < 0.001; HR 1.17 [95% CI, 1.01–1.37], *p* = 0.038). However, the pairwise comparison of patients with IDDM to patients with NIDDM showed significantly higher rates of cardiac death (HR 1.35 [95% CI, 1.08–1.69], *p* = 0.009), all-cause death (HR 1.35 [95% CI, 1.12–1.63], *p* = 0.002), and MI (HR 1.38 [95% CI, 1.02–1.89], *p* = 0.039) in insulin-treated diabetics but comparable revascularization rates in both diabetic groups (TLR: HR 1.16 [95% CI, 0.94–1.43], *p* = 0.157). Clinical outcomes of patients with IDDM, NIDDM, and non-diabetics are shown in detail in Table 4. Event curves for cardiac death, TLR, and MI based on the subdivision of patients with IDDM, NIDDM, and nondiabetics are presented in Figures S3-S5. To assess the influence of the treatment modality on outcomes over the entire period of study inclusion, we divided the total patient cohort into deciles based on the time of first PCI of DES-ISR. After 3 years, the *p* values for interaction between DM and the time of first PCI of DES-ISR did not reveal significant differences either for cardiac death or for TLR. Moreover, after 3 years, the *p* values for interaction between DM and type of intervention (DES vs. DCB vs. POBA) did not show significant differences either for cardiac death or for TLR (Table S3 and Figure S6).

**Table 4:** Clinical outcomes (IDDM vs. NIDDM vs. nondiabetics)

	PCI in IDDM (*n* = 471)	PCI in NIDDM (*n* = 771)	PCI in NON (*n* = 2269)	HR IDDM-NON (95% CI)	*p* value	HR NIDDM-NON (95% CI)	*p* value	HR IDDM-NIDDM (95% CI)	*p* value
All-cause death	187 (62.8)	255 (54.7)	622 (43.4)	1.91 (1.62–2.25)	< 0.001	1.41 (1.22–1.63)	< 0.001	1.35 (1.12–1.63)	0.002
Cardiac death	134 (47.6)	184 (40.0)	458 (32.8)	1.88 (1.55–2.28)	< 0.001	1.39 (1.17–1.65)	< 0.001	1.35 (1.08–1.69)	0.009
MI	72 (18.8)	92 (14.1)	191 (9.7)	2.17 (1.65–2.84)	< 0.001	1.57 (1.22–2.01)	< 0.001	1.38 (1.02–1.89)	0.039
TLR	148 (34.9)	227 (32.6)	613 (29.4)	1.37 (1.14–1.64)	< 0.001	1.17 (1.01–1.37)	0.038	1.16 (0.94–1.43)	0.157
TVR	124 (29.2)	206 (30.3)	515 (25.2)	1.38 (1.13–1.69)	0.001	1.30 (1.10–1.52)	0.002	1.07 (0.85–1.34)	0.580
NTVR	182 (43.7)	301 (43.6)	737 (36.4)	1.41 (1.20–1.65)	< 0.001	1.37 (1.20–1.56)	< 0.001	1.03 (0.86–1.23)	0.772
Definite ST	12 (2.7)	15 (2.1)	35 (1.6)	1.81 (0.94–3.49)	0.075	1.33 (0.73–2.44)	0.351	1.36 (0.64–2.91)	0.427

The numbers shown in brackets are Kaplan–Meier estimates (%). Cumulative incidence functions were computed for outcomes other than death to account for competing risks. The adjusted hazard ratios, 95% CI, and *P* values reported here are derived from a conventional multivariable analysis with adjustment for the following variables: age, sex, multivessel CAD, prior MI, prior CABG, ejection fraction, use of DCB, Creatinine and C-reactive protein ad admission, acetylsalicylic acid, P2Y12 inhibitor and statin at discharge

Target vessel revascularization is defined as revascularization in the target vessel outside the target lesion

*CABG*, coronary artery bypass graft; *CAD*, coronary artery disease; *DCB*, drug-coated balloon; *HR*, hazard ratio; *HR*_adj_, adjusted hazard ratio; *IDDM*, insulin-dependent diabetes mellitus; *MI*, myocardial infarction; *NIDDM*, non-insulin-dependent diabetes mellitus; *NON*, nondiabetics; *NTVR*, nontarget vessel revascularization; *ST*, stent thrombosis; *TLR*, target lesion revascularization; *TVR*, target vessel revascularization

### Landmark analyses

**Fig. 3:**
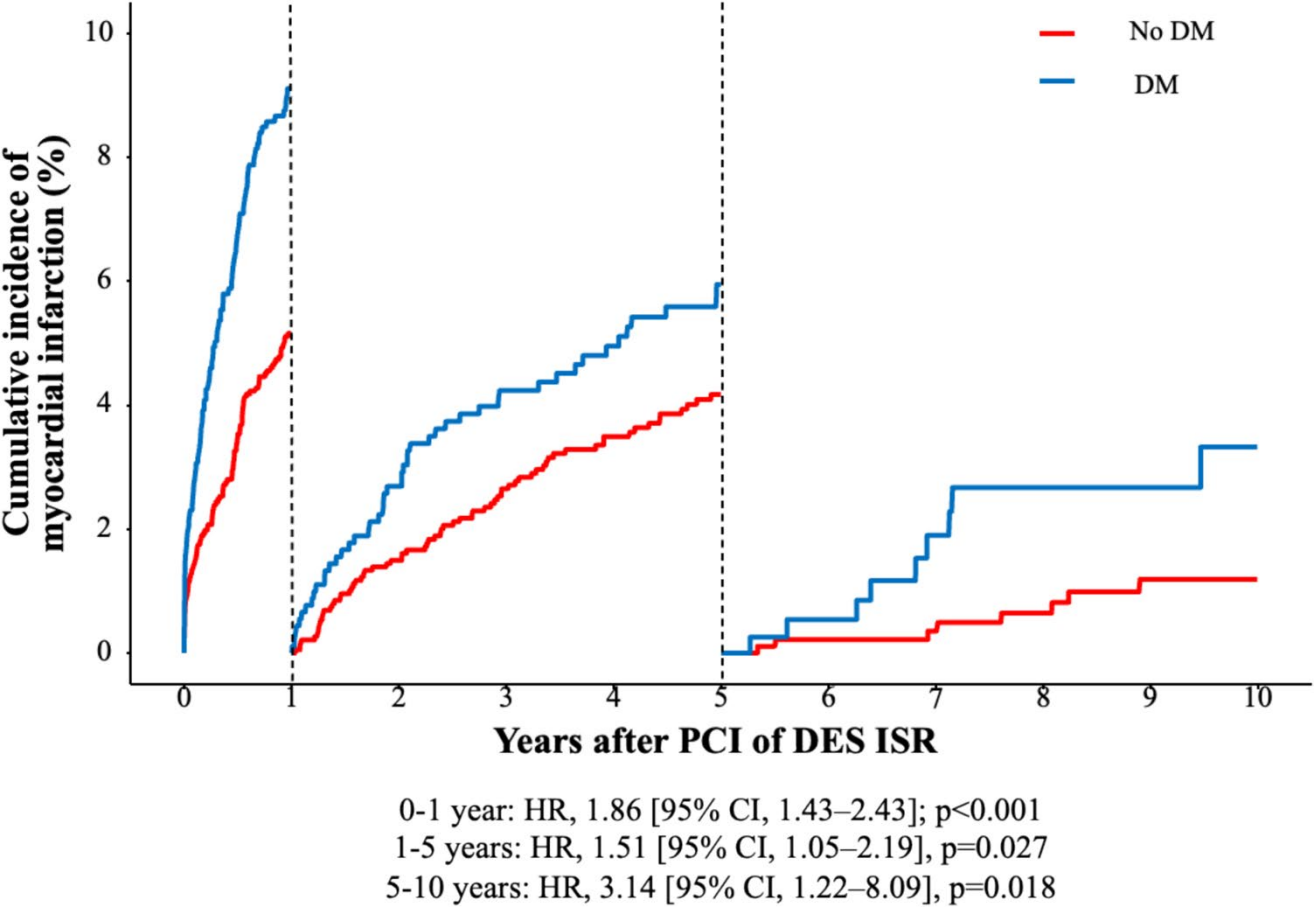
Landmark analysis of MI by diabetes. The figure demonstrates the cumulative incidence function curve and HR with accompanying 95% CI. Abbreviations: DES, drug-eluting stent; DM, diabetes mellitus; HR, hazard ratio; ISR, in-stent restenosis; MI, myocardial infarction; PCI, percutaneous coronary intervention; TLR, target lesion revascularization

Cardiac death occurred in 286 of 3511 patients within the first year after PCI of DES-ISR, with an increased risk in the diabetic group (HR, 1.93 [95% CI, 1.46–2.55], *p* < 0.001). From one to five years after PCI, cardiac death occurred in 512 of 2925 patients, with an increased risk in the diabetic group (HR, 1.54 [95% CI, 1.25–1.89], *p* < 0.001). From five to ten years after PCI, cardiac death occurred in 266 of 1449 patients and was comparable in the two groups (HR, 1.30 [95% CI, 0.98–1.73], *p* = 0.072). These results are demonstrated in Figure S7. TLR occurred in 941 of 3511 patients within the first year after PCI of DES-ISR, with an increased risk in diabetics (HR, 1.25 [95% CI, 1.07–1.44], *p* = 0.004). From one to five years after PCI, TLR occurred in 614 of 2411 patients and was comparable in both groups (HR, 1.17 [95% CI, 0.89–1.54], *p* = 0.248). From five to ten years after PCI, TLR occurred in 230 of 1159 patients, with comparable rates in the two groups (HR, 2.00 [95% CI, 0.92–4.33], *p* = 0.078). These results are demonstrated in Figure S8. MI occurred in 457 of 3511 patients within the first year after PCI of DES-ISR and was more frequent in the diabetic group (HR, 1.86 [95% CI, 1.43–2.43]; *p* < 0.001). MI occurred in 562 of 2815 patients from one to five years after PCI, with an increased risk in diabetics (HR, 1.51 [95% CI, 1.05–2.19], *p* = 0.027). From five to ten years after PCI, MI occurred in 262 of 1375 patients, with increased MI rates in diabetic patients (HR, 3.14 [95% CI, 1.22–8.09], *p* = 0.018). These results are demonstrated in Fig. 3.

### Predictors of TLR

Older patient age and current smokers were found to be preventive parameters of TLR after 10 years. Moreover, DES implantation in comparison to either balloon angioplasty (BA) or DCB was found to be a preventive factor of TLR after 10 years. However, arterial hypertension, multivessel CAD, ACS, and prior CABG were found to be associated with a higher likelihood of TLR after 10 years. A summary of predictors of TLR is shown in Table 5.

**Table 5:** Predictors of TLR after PCI of first DES-ISR

	HR (95% CI)	*p* value
Older age	0.98 (0.98–0.99)	< 0.001
Female sex	1.00 (0.87–1.15)	0.988
BMI	1.00 (0.99–1.02)	0.528
Arterial hypertension	1.37 (1.03–1.82)	0.030
Hypercholesterinemia	0.99 (0.87–1.13)	0.938
Current smoker	0.82 (0.70–0.97)	0.018
Multivessel CAD	1.43 (1.14–1.79)	0.002
ACS	1.21 (1.08–1.36)	0.001
Prior MI	1.06 (0.95–1.18)	0.311
Prior CABG	1.29 (1.11–1.51)	< 0.001
Ejection fraction	1.00 (1.00–1.01)	0.605
DES implantation	0.71 (0.63–0.80)	< 0.001
Drug-coated balloon	0.98 (0.84–1.15)	0.836
Ostial lesion	1.14 (0.99–1.30)	0.060
Bifurcation lesion	0.91 (0.80–1.04)	0.167

*ACS*, acute coronary syndrome; *BMI*, body mass index; *CABG*, coronary artery bypass graft; *CAD*, coronary artery disease; *DES*, drug-eluting stent; *ISR*, in-stent restenosis; *MI*, myocardial infarction; *PCI*, percutaneous coronary intervention; *TLR*, target lesion revascularization

### Prediction of recurrent TLR

Diabetics are predisposed to a significantly higher risk of recurrent TLR compared to nondiabetics (HR 1.20 [95% CI, 1.07–1.33], *p* = 0.001). By dividing the diabetic group into patients with IDDM and patients with NIDDM, one can recognize that the risk of recurrent TLR is significantly higher in patients with IDDM than in non-diabetics (HR 1.26 [95% CI, 1.09–1.47], p = 0.001) and significantly higher in patients with NIDDM than in nondiabetics (HR 1.16 [95% CI, 1.02–1.31], *p* = 0.027). Patients with IDDM show slightly higher rates of recurrent TLR than patients with NIDDM without reaching the level of significance (HR 1.09 [95% CI, 0.92–1.30], *p* = 0.308).

## Discussion

This analysis addresses the limited data concerning long-term outcomes after PCI of DES-ISR in patients with and without DM. The major findings are as follows:

- Ten-year cardiac death rate is higher in diabetics than in nondiabetics.
- At ten-year follow-up, the MI rate is higher in diabetics than in nondiabetics.
- Diabetics were found out to have higher rates of repeat revascularization of target lesion, target vessel and non-target vessel than nondiabetics in the long-term.
- Patients with IDDM show significantly higher rates of cardiac death, MI, and TLR than nondiabetics, and higher rates of cardiac death and MI than patients with NIDDM, with comparable revascularization rates in both diabetic groups.
- Arterial hypertension, multivessel CAD, ACS, and prior CABG were found to be predictive parameters of TLR after ten years in patients with DM.

DM is a well-known cardiovascular risk factor increasing the risk of adverse events among patients suffering from CAD and undergoing PCI with DES [9, 22–24]. According to follow-up data from the Swedish Coronary Angiography and Angioplasty Registry, mortality rates after PCI are higher in diabetics than in nondiabetics in the long term, with an increasing mortality gap with follow-up time [25].

On the one hand, DM predisposes patients to a higher risk of ISR after PCI in de novo lesions [26, 27]; on the other hand, PCI of ISR implicates higher rates of adverse events than PCI in de novo lesions [6]. Previously published trials found that hyperglycemia at admission predicts second-generation DES-ISR after one year [28], insulin resistance is closely linked to the rates of DES-ISR [29, 30], and hyperinsulinemia is an independent risk factor for ISR [31, 32]. So, diabetics with DES-ISR face “double jeopardy” with DM as a well-known cardiovascular risk factor considerably predicting adverse outcomes on the one hand, and with the occurrence of ISR strongly associated with worse outcomes on the other hand. A previously published registry exploring trends of PCI for ISR in the United States emphasized the high prevalence of DM in patients with DES-ISR, with 48.5% suffering from DM [4].

Previously published data analyzing clinical outcomes after PCI for ISR revealed increased prevalence of higher body mass index (BMI), hypertension, and hyperlipidemia in diabetics as compared to nondiabetics [16, 23], congruent with data from our analysis. Consistent with the results from the underlying trial, Tanner et al. figured out diabetics to be more often female than nondiabetics [16]. Regarding clinical presentation, previously published data found comparable rates between diabetics and nondiabetics [14, 16, 17]. Based on data from the underlying trial, more than 30% of diabetics with DES-ISR present with ACS, underlining the clinical importance of DM in patients suffering from CAD. ACS was revealed to be predictive of TLR in the long term, consistent with previously published data [16, 17]. Based on our analysis, diabetics more often underwent prior CABG, concordant with previous data and underlining the severity of CAD in diabetics [16, 22]. Moreover, prior CABG was found to be predictive of TLR in the long term, consistent with one-year follow-up in a previously published registry [16]. According to higher rates of chronic kidney disease in patients with DM reported by Tanner et al., the underlying trial showed higher values of creatinine at admission in diabetics [16].

To our knowledge, the underlying trial is the first analysis including a large cohort of patients treated for DES-ISR and followed-up long-term for clinical outcomes based on diabetic status. Previously published trials with similar patient cohorts have conducted follow-up for a maximum of two years [14, 16, 17]. Tanner et al. demonstrated DM to be associated with poorer one-year clinical outcomes in terms of higher rates of major adverse cardiac events (MACE) consisting of all-cause death, MI and TVR in 3156 patients undergoing PCI for ISR [16]; congruent with worse long-term outcomes in diabetics reported in the underlying trial. However, Zhao et al. figured out MACE consisting of cardiac death, MI and TLR to be similar in patients with and without DM after two years [14]. Paramasivam et al. did not reveal relevant differences in one-year clinical outcomes focusing on all-cause death, MI and TLR between diabetics and nondiabetics [17]. In both analyses, the authors conclude DM to not be a factor associated with poor clinical outcomes based on currently available treatments. However, besides the fact that both trials solely included 81 and 109 diabetics, follow-up duration was shorter than in the underlying trial [14, 17]. In the underlying trial landmark, analyses emphasize the consistently over ten years increased risk of MI for diabetics while the elevated risk of cardiac death for diabetics particularly exists in the first five years after PCI of DES-ISR. The increased risk of TLR in diabetics in the long-term appears to be caused by increased TLR rates in the first year after PCI of DES-ISR with largely the same risk in both groups in the further long-term.

Consistent with the results from our analysis, the occurrence of ISR was found to be more frequent in patients suffering from DM, with even greater rates in patients with IDDM [33]. Moreover, our analysis revealed higher rates of cardiac death, all-cause death, and MI after PCI of DES-ISR in patients with IDDM than in patients with NIDDM. Based on our results, rates of repeat revascularization did not show relevant differences between both diabetic groups. Konigstein et al. investigated the impact of insulin dependence on one- and two-year clinical outcomes after PCI in patients suffering from DM and found that TVR and target lesion failure, consisting of cardiac death, target vessel MI, and ischemia-driven TLR, were more frequent in insulin-dependent diabetics [23]. Pi et al. revealed cardiac death, any revascularization, and ST to be more often present in patients with IDDM than in patients with NIDDM [34]. Biswas et al. found an increase in adverse events with the escalation of antidiabetic treatment from diet control to oral antidiabetic drug treatment to insulin treatment [35]. However, all three trials analyzed outcomes after PCI of de novo coronary lesions [23, 34, 35].

Development of ISR is particularly based on neointimal hyperplasia, which is characterized by small muscle cell proliferation and typically emerges soon after PCI, and on neo-atherosclerosis, which implicates the formation of lipid-rich macrophages within the neointima and usually appears after a minimum of one year [36–39]. Besides, the link between DM and ISR is based on endothelial dysfunction, increased inflammatory response, and the coexistence of comorbidities [39]. Diabetics tend to present with more severe atherosclerotic burden, smaller reference vessel diameter, and less collateral perfusion, making the PCI more challenging than in nondiabetics [23, 40, 41]. Moreover, CAD in diabetics often comprises long and diffuse diseased coronary segments with a higher burden of calcification and distinct tortuosity[42]. As implanted DES do not rarely land in diseased segments, diabetics are predisposed to edge restenosis, which could partially be avoided by routine use of intravascular imaging pre-PCI [17]. So optimal stent expansion and gaining adequate minimum luminal area are required to avoid or at least delay the onset of ISR [16, 43]. Previously published data suggested the strong prediction of TLR and TVR in diabetics to be attributed to complex coronary lesions and to a decrease in simple coronary lesions [9].

According to recently published guidelines of the European Society of Cardiology (ESC), DES are recommended over DCB for the treatment of DES-ISR based on superior antirestenotic efficacy [44]. After PCI of DES-ISR, the DAEDALUS trial showed three-year TLR rates to be significantly lower with repeat DES treatment than with DCB [45]. Ten-year outcomes of the ISAR-DESIRE 3 trial revealed comparative results between treatment with DES and DCB in DES-ISR, with numerically higher TLR rates in DCB-treated patients; however, the threshold for statistical significance was not reached [46]. Based on our data, diabetics less frequently underwent DES implantation and more frequently received DCB for the treatment of DES-ISR than nondiabetics. This therapeutic approach might be a potential confounder, as previously published data revealed comparable MACE rates, significantly improved survival, and a modest increase in TLR rates in diabetics with DES-ISR or de novo lesions who underwent DCB treatment in comparison to repeat DES treatment [47].

In addition to constant progress in medical and interventional strategies for the optimal treatment of CAD, antidiabetic drug treatment, diet control, and lifestyle modification play a decisive role in secondary prevention to effectively slow down the progression of CAD in patients with DM. Newer generation DES with thinner struts and coated with new antiproliferative drugs, with the aid of more routinely used intravascular imaging, might further improve long-term safety, go towards reduced rates of adverse events, and thus represent a suitable treatment option for a wider range of diabetics with DES-ISR in the future.

### Limitations

The results of our analysis should be interpreted with caution as there are several limitations. First, it is an observational retrospective study and so is subject to all limitations of this design. Thus, some information is not available for every patient, e.g. exact cause of death, data on glycemic control (e.g., HbA1c), details of index PCI, or selected procedural characteristics of patients who were initially treated elsewhere. Second, substantial advances in interventional treatment have been made with the implementation of DCB and newer generation DES with new antiproliferative drugs and improved stent structure during the inclusion period. In comparison to early-generation paclitaxel- or sirolimus-coated DES, the implantation of newer generation everolimus- or zotarolimus-coated DES resulted in reduced rates of ISR and ST in the long term. So, the comparison of both treatment strategies (DCB vs. DES) over time is only possible to a limited extent. Third, progress in the dual antiplatelet therapy regime based on the optimal balance between prevention of ischemic events on the one hand and bleeding events on the other hand has been made during the last decades. Fourth, the application of intravascular imaging has revolutionized the interventional treatment strategies of CAD. Intravascular imaging is an important and increasingly used tool for better understanding the underlying pathomechanism of ISR and so for applying the optimal interventional therapeutic approach. However, intravascular imaging has not been systematically applied in the present trial. Fifth, as both DES and DCB were equally recommended for treatment of DES-ISR for years, the therapeutic approach in DES-ISR treatment might particularly be driven by individual experiences in applying DES and DCB treatment for every interventionalist.

## Conclusions

Despite advances in medical and interventional treatment strategies, long-term rates of mortality and adverse events after PCI of DES-ISR are high among patients suffering from DM. Diabetics, especially patients with IDDM, show higher rates of cardiac death, of MI, and of repeat revascularization of target lesion, target vessel, and nontarget vessel compared to nondiabetics ten years after ISR PCI. Based on a growing prevalence of DM worldwide on the one hand and the close link of DM and ISR on the other hand, the need to further investigate diabetes-related long-term outcomes after PCI of DES-ISR appears increasingly important.

## Supplementary Information

Below is the link to the electronic supplementary material.

ESM 1 (DOCX 557 KB)

## Data Availability

The data that support the findings of this study are available from the ISAResearch-Center, Munich, Germany.

## Abbreviations

ACS: Acute coronary syndrome
BA: Balloon angioplasty
CABG: Coronary artery bypass graft
CAD: Coronary artery disease
DCB: Drug-coated balloon
DES: Drug-eluting stent
DM: Diabetes mellitus
HR: Hazard ratio
IDDM: Insulin-dependent diabetes mellitus
ISR: In-stent restenosis
MACE: Major adverse cardiac events
MI: Myocardial infarction
NIDDM: Non-insulin-dependent diabetes mellitus
NTVR: Nontarget vessel revascularization
PCI: Percutaneous coronary intervention
ST: Stent thrombosis
TLR: Target lesion revascularization
TVR: Target vessel revascularization

## References

[1] Duband B, Souteyrand G, Clerc JM, Chassaing S, Fichaux O, Marcollet P, Deballon R, Roussel L, Pereira B, Collet JP et al (2023) Prevalence, management and outcomes of percutaneous coronary intervention for coronary in-stent restenosis: insights from the France PCI registry. Cardiovasc Revasc Med 52:39–46. 10.1016/j.carrev.2023.02.006 36813696 10.1016/j.carrev.2023.02.006

[2] Madhavan MV, Kirtane AJ, Redfors B, Genereux P, Ben-Yehuda O, Palmerini T, Benedetto U, Biondi-Zoccai G, Smits PC, von Birgelen C et al (2020) Stent-related adverse events >1 year after percutaneous coronary intervention. J Am Coll Cardiol 75:590–604. 10.1016/j.jacc.2019.11.058 32057373 10.1016/j.jacc.2019.11.058

[3] Cassese S, Byrne RA, Schulz S, Hoppman P, Kreutzer J, Feuchtenberger A, Ibrahim T, Ott I, Fusaro M, Schunkert H et al (2015) Prognostic role of restenosis in 10 004 patients undergoing routine control angiography after coronary stenting. Eur Heart J 36:94–99. 10.1093/eurheartj/ehu383 25298237 10.1093/eurheartj/ehu383

[4] Moussa ID, Mohananey D, Saucedo J, Stone GW, Yeh RW, Kennedy KF, Waksman R, Teirstein P, Moses JW, Simonton C (2020) Trends and outcomes of restenosis after coronary stent implantation in the United States. J Am Coll Cardiol 76:1521–1531. 10.1016/j.jacc.2020.08.002 32972528 10.1016/j.jacc.2020.08.002

[5] Neumann FJ, Sousa-Uva M, Ahlsson A, Alfonso F, Banning AP, Benedetto U, Byrne RA, Collet JP, Falk V, Head SJ et al (2018) 2018 ESC/EACTS guidelines on myocardial revascularization. Eur Heart J 40(2):87–165. 10.1093/eurheartj/ehy394 10.1093/eurheartj/ehy394 30165437

[6] Elbadawi A, Dang AT, Mahana I, Elzeneini M, Alonso F, Banerjee S, Kumbhani DJ, Elgendy IY, Mintz GS (2023) Outcomes of percutaneous coronary intervention for in-stent restenosis versus de novo lesions: a meta-analysis. J Am Heart Assoc 12:e029300. 10.1161/JAHA.122.029300 37382147 10.1161/JAHA.122.029300 PMC10356080

[7] Sun H, Saeedi P, Karuranga S, Pinkepank M, Ogurtsova K, Duncan BB, Stein C, Basit A, Chan JCN, Mbanya JC et al (2022) IDF diabetes atlas: global, regional and country-level diabetes prevalence estimates for 2021 and projections for 2045. Diabetes Res Clin Pract 183:109119. 10.1016/j.diabres.2021.109119 34879977 10.1016/j.diabres.2021.109119 PMC11057359

[8] Aronson D, Rayfield EJ (2002) How hyperglycemia promotes atherosclerosis: molecular mechanisms. Cardiovasc Diabetol 1:1. 10.1186/1475-2840-1-1 12119059 10.1186/1475-2840-1-1 PMC116615

[9] Kedhi E, Genereux P, Palmerini T, McAndrew TC, Parise H, Mehran R, Dangas GD, Stone GW (2014) Impact of coronary lesion complexity on drug-eluting stent outcomes in patients with and without diabetes mellitus: analysis from 18 pooled randomized trials. J Am Coll Cardiol 63:2111–2118. 10.1016/j.jacc.2014.01.064 24632279 10.1016/j.jacc.2014.01.064

[10] Kapur A, Hall RJ, Malik IS, Qureshi AC, Butts J, de Belder M, Baumbach A, Angelini G, de Belder A, Oldroyd KG, et al (2010) Randomized comparison of percutaneous coronary intervention with coronary artery bypass grafting in diabetic patients. 1-year results of the CARDia (Coronary Artery Revascularization in Diabetes) trial. J Am Coll Cardiol. 55:432–440. 10.1016/j.jacc.2009.10.014 10.1016/j.jacc.2009.10.014 20117456

[11] Billinger M, Raber L, Hitz S, Stefanini GG, Pilgrim T, Stettler C, Zanchin T, Pulver C, Pfaffli N, Eberli F et al (2012) Long-term clinical and angiographic outcomes of diabetic patients after revascularization with early generation drug-eluting stents. Am Heart J 163:876. 10.1016/j.ahj.2012.02.014 22607867 10.1016/j.ahj.2012.02.014

[12] Iijima R, Ndrepepa G, Mehilli J, Markwardt C, Bruskina O, Pache J, Ibrahim M, Schomig A, Kastrati A (2007) Impact of diabetes mellitus on long-term outcomes in the drug-eluting stent era. Am Heart J 154:688–693. 10.1016/j.ahj.2007.06.005 17892992 10.1016/j.ahj.2007.06.005

[13] Alfonso F, Byrne RA, Rivero F, Kastrati A (2014) Current treatment of in-stent restenosis. J Am Coll Cardiol 63:2659–2673. 10.1016/j.jacc.2014.02.545 10.1016/j.jacc.2014.02.545 24632282

[14] Zhao L, Zhu W, Zhang X, He D, Guo C (2017) Effect of diabetes mellitus on long-term outcomes after repeat drug-eluting stent implantation for in-stent restenosis. BMC Cardiovasc Disord 17:16. 10.1186/s12872-016-0445-6 28061808 10.1186/s12872-016-0445-6 PMC5217259

[15] Mohebi R, Chen C, Ibrahim NE, McCarthy CP, Gaggin HK, Singer DE, Hyle EP, Wasfy JH, Januzzi JL Jr. (2022) Cardiovascular disease projections in the United States based on the 2020 census estimates. J Am Coll Cardiol 80:565–578. 10.1016/j.jacc.2022.05.033 10.1016/j.jacc.2022.05.033 PMC9396356 35926929

[16] Tanner R, Farhan S, Giustino G, Sartori S, Feng Y, Hooda A, Vinayak M, Dangas G, Mehran R, Kini AS et al (2024) Impact of diabetes mellitus on clinical outcomes after first episode in-stent restenosis PCI: results from a large registry. Int J Cardiol 401:131856. 10.1016/j.ijcard.2024.131856 38360097 10.1016/j.ijcard.2024.131856

[17] Paramasivam G, Devasia T, Jayaram A, U KA, Rao MS, Vijayvergiya R, Nayak K (2020) In-stent restenosis of drug-eluting stents in patients with diabetes mellitus: clinical presentation, angiographic features, and outcomes. Anatol J Cardiol 23:28–34 31911567 10.14744/AnatolJCardiol.2019.72916 PMC7141436

[18] Expert Committee on the D, Classification of Diabetes M (2003) Report of the expert committee on the diagnosis and classification of diabetes mellitus. Diabetes Care. 26 Suppl 1:S5–20. 10.2337/diacare.26.2007.s5 10.2337/diacare.26.2007.s5 12502614

[19] Thygesen K, Alpert JS, Jaffe AS, Simoons ML, Chaitman BR, White HD, Joint ESCAAHAWHFTFftUDoMI, Katus HA, Lindahl B, Morrow DA, et al. (2012) Third universal definition of myocardial infarction. Circulation. 126:2020–2035. 10.1161/CIR.0b013e31826e1058 10.1161/CIR.0b013e31826e1058 22923432

[20] Cutlip DE, Windecker S, Mehran R, Boam A, Cohen DJ, van Es GA, Steg PG, Morel MA, Mauri L, Vranckx P et al (2007) Clinical end points in coronary stent trials: a case for standardized definitions. Circulation 115:2344–2351. 10.1161/CIRCULATIONAHA.106.685313 17470709 10.1161/CIRCULATIONAHA.106.685313

[21] Garcia-Garcia HM, McFadden EP, Farb A, Mehran R, Stone GW, Spertus J, Onuma Y, Morel MA, van Es GA, Zuckerman B et al (2018) Standardized end point definitions for coronary intervention trials: the Academic Research Consortium-2 consensus document. Circulation 137:2635–2650. 10.1161/CIRCULATIONAHA.117.029289 29891620 10.1161/CIRCULATIONAHA.117.029289

[22] Aronson D, Edelman ER (2014) Coronary artery disease and diabetes mellitus. Cardiol Clin 32:439–455. 10.1016/j.ccl.2014.04.001 25091969 10.1016/j.ccl.2014.04.001 PMC4672945

[23] Konigstein M, Ben-Yehuda O, Smits PC, Love MP, Banai S, Perlman GY, Golomb M, Ozan MO, Liu M, Leon MB et al (2018) Outcomes among diabetic patients undergoing percutaneous coronary intervention with contemporary drug-eluting stents: analysis from the BIONICS randomized trial. JACC Cardiovasc Interv 11:2467–2476. 10.1016/j.jcin.2018.09.033 30573057 10.1016/j.jcin.2018.09.033

[24] Koskinas KC, Siontis GC, Piccolo R, Franzone A, Haynes A, Rat-Wirtzler J, Silber S, Serruys PW, Pilgrim T, Raber L et al (2016) Impact of diabetic status on outcomes after revascularization with drug-eluting stents in relation to coronary artery disease complexity: patient-level pooled analysis of 6081 patients. Circ Cardiovasc Interv 9:e003255. 10.1161/CIRCINTERVENTIONS.115.003255 26823484 10.1161/CIRCINTERVENTIONS.115.003255

[25] Norhammar A, Lagerqvist B, Saleh N (2010) Long-term mortality after PCI in patients with diabetes mellitus: results from the Swedish Coronary Angiography and Angioplasty Registry. EuroIntervention 5:891–897 20542773

[26] Qin SY, Zhou Y, Jiang HX, Hu BL, Tao L, Xie MZ (2013) The association of diabetes mellitus with clinical outcomes after coronary stenting: a meta-analysis. PLoS ONE 8:e72710. 10.1371/journal.pone.0072710 24066025 10.1371/journal.pone.0072710 PMC3774683

[27] Arnold SV, Bhatt DL, Barsness GW, Beatty AL, Deedwania PC, Inzucchi SE, Kosiborod M, Leiter LA, Lipska KJ, Newman JD et al (2020) Clinical management of stable coronary artery disease in patients with type 2 diabetes mellitus: a scientific statement from the American Heart Association. Circulation 141:e779–e806. 10.1161/CIR.0000000000000766 32279539 10.1161/CIR.0000000000000766 PMC12204403

[28] Mone P, Gambardella J, Minicucci F, Lombardi A, Mauro C, Santulli G (2021) Hyperglycemia drives stent restenosis in STEMI patients. Diabetes Care 44:e192–e193. 10.2337/dc21-0939 34531311 10.2337/dc21-0939 PMC8546275

[29] Zhao LP, Xu WT, Wang L, Li H, Shao CL, Gu HB, Chan SP, Xu HF, Yang XJ (2015) Influence of insulin resistance on in-stent restenosis in patients undergoing coronary drug-eluting stent implantation after long-term angiographic follow-up. Coron Artery Dis 26:5–10. 10.1097/MCA.0000000000000170 25211654 10.1097/MCA.0000000000000170

[30] Hong SJ, Kim MH, Ahn TH, Ahn YK, Bae JH, Shim WJ, Ro YM, Lim DS (2006) Multiple predictors of coronary restenosis after drug-eluting stent implantation in patients with diabetes. Heart 92:1119–1124. 10.1136/hrt.2005.075960 16449516 10.1136/hrt.2005.075960 PMC1861125

[31] Imazu M, Sumii K, Yamamoto H, Toyofuku M, Okimoto T, Gomyo Y, Ueda H, Hayashi Y, Kohno N (2001) Hyperinsulinemia as a risk factor for restenosis after coronary balloon angioplasty. Jpn Circ J 65:947–952. 10.1253/jcj.65.947 11716244 10.1253/jcj.65.947

[32] Aronson D (1996) Restenosis in diabetic patients: is hyperinsulinemia the culprit? Circulation 94:3003–3005 8941148

[33] Gilbert J, Raboud J, Zinman B (2004) Meta-analysis of the effect of diabetes on restenosis rates among patients receiving coronary angioplasty stenting. Diabetes Care 27:990–994. 10.2337/diacare.27.4.990 15047662 10.2337/diacare.27.4.990

[34] Pi SH, Rhee TM, Lee JM, Hwang D, Park J, Park TK, Yang JH, Song YB, Choi JH, Hahn JY et al (2018) Outcomes in patients with diabetes mellitus according to insulin treatment after percutaneous coronary intervention in the second-generation drug-eluting stent era. Am J Cardiol 121:1505–1511. 10.1016/j.amjcard.2018.02.034 29751955 10.1016/j.amjcard.2018.02.034

[35] Biswas S, Dinh D, Andrianopoulos N, Lefkovits J, Ajani A, Duffy SJ, Chan W, Walton A, Brennan A, Clark DJ et al (2021) Comparison of long-term outcomes after percutaneous coronary intervention in patients with insulin-treated versus non-insulin treated diabetes mellitus. Am J Cardiol 148:36–43. 10.1016/j.amjcard.2021.02.025 33667454 10.1016/j.amjcard.2021.02.025

[36] Virmani R, Farb A (1999) Pathology of in-stent restenosis. Curr Opin Lipidol 10:499–506. 10.1097/00041433-199912000-00004 10680043 10.1097/00041433-199912000-00004

[37] Otsuka F, Byrne RA, Yahagi K, Mori H, Ladich E, Fowler DR, Kutys R, Xhepa E, Kastrati A, Virmani R et al (2015) Neoatherosclerosis: overview of histopathologic findings and implications for intravascular imaging assessment. Eur Heart J 36:2147–2159. 10.1093/eurheartj/ehv205 25994755 10.1093/eurheartj/ehv205

[38] Marazzi G, Wajngarten M, Vitale C, Patrizi R, Pelliccia F, Gebara O, Pierri H, Ramires JA, Volterrani M, Fini M et al (2007) Effect of free fatty acid inhibition on silent and symptomatic myocardial ischemia in diabetic patients with coronary artery disease. Int J Cardiol 120:79–84. 10.1016/j.ijcard.2006.08.082 17134770 10.1016/j.ijcard.2006.08.082

[39] Pelliccia F, Zimarino M, Niccoli G, Morrone D, De Luca G, Miraldi F, De Caterina R (2023) In-stent restenosis after percutaneous coronary intervention: emerging knowledge on biological pathways. Eur Heart J Open 3:oead083. 10.1093/ehjopen/oead083 37808526 10.1093/ehjopen/oead083 PMC10558044

[40] Jensen LO, Thayssen P, Mintz GS, Maeng M, Junker A, Galloe A, Christiansen EH, Hoffmann SK, Pedersen KE, Hansen HS et al (2007) Intravascular ultrasound assessment of remodelling and reference segment plaque burden in type-2 diabetic patients. Eur Heart J 28:1759–1764. 10.1093/eurheartj/ehm175 17540850 10.1093/eurheartj/ehm175

[41] Abaci A, Oguzhan A, Kahraman S, Eryol NK, Unal S, Arinc H, Ergin A (1999) Effect of diabetes mellitus on formation of coronary collateral vessels. Circulation 99:2239–2242. 10.1161/01.cir.99.17.2239 10226087 10.1161/01.cir.99.17.2239

[42] Bernelli C, Chan J, Chieffo A (2014) Drug-eluting stent outcomes in diabetes. Expert Rev Cardiovasc Ther 12:95–109. 10.1586/14779072.2014.853615 24345095 10.1586/14779072.2014.853615

[43] Giustino G, Colombo A, Camaj A, Yasumura K, Mehran R, Stone GW, Kini A, Sharma SK (2022) Coronary in-stent restenosis: JACC state-of-the-art review. J Am Coll Cardiol 80:348–372. 10.1016/j.jacc.2022.05.017 35863852 10.1016/j.jacc.2022.05.017

[44] Vrints C, Andreotti F, Koskinas KC, Rossello X, Adamo M, Ainslie J, Banning AP, Budaj A, Buechel RR, Chiariello GA et al (2024) 2024 ESC guidelines for the management of chronic coronary syndromes. G Ital Cardiol (Rome) 25:e1–e132. 10.1714/4375.43725 39611224 10.1714/4375.43725

[45] Giacoppo D, Alfonso F, Xu B, Claessen B, Adriaenssens T, Jensen C, Perez-Vizcayno MJ, Kang DY, Degenhardt R, Pleva L et al (2020) Drug-coated balloon angioplasty versus drug-eluting stent implantation in patients with coronary stent restenosis. J Am Coll Cardiol 75:2664–2678. 10.1016/j.jacc.2020.04.006 32466881 10.1016/j.jacc.2020.04.006

[46] Giacoppo D, Alvarez-Covarrubias HA, Koch T, Cassese S, Xhepa E, Kessler T, Wiebe J, Joner M, Hochholzer W, Laugwitz KL et al (2023) Coronary artery restenosis treatment with plain balloon, drug-coated balloon, or drug-eluting stent: 10-year outcomes of the ISAR-DESIRE 3 trial. Eur Heart J 44:1343–1357. 10.1093/eurheartj/ehad026 36807512 10.1093/eurheartj/ehad026

[47] Verdoia M, Zilio F, Gioscia R, Viola O, Brancati MF, Fanti D, Solda PL, Bonmassari R, Rognoni A, De Luca G (2023) Prognostic impact of drug-coated balloons in patients with diabetes mellitus: a propensity-matched study. Am J Cardiol 206:73–78. 10.1016/j.amjcard.2023.08.113 37683582 10.1016/j.amjcard.2023.08.113

