# Supplementary material for: Diabetes-related ten-year outcomes after percutaneous coronary intervention of in-stent restenosis
Source: Clin Res Cardiol. 2025 Nov 3;115(1):132–44. doi: 10.1007/s00392-025-02782-6 (PMC12783287; doi:10.1007/s00392-025-02782-6)

**SUPPLEMENTAL MATERIAL**

**Tables
Table S1.** Clinical baseline characteristics (IDDM vs. NIDDM vs. non-diabetics)
**Table S2.** Procedural baseline characteristics (IDDM vs. NIDDM vs. non-diabetics)
**Table S3.** Three-year outcomes in time deciles based on time of first PCI of DES-ISR **Figures
Figure S1.** Type of treatment depending on time of PCI of DES-ISR **Figure S2.** Diabetes-related ten-year cumulative incidence of MI **Figure S3.** Diabetes-related ten-year incidence of cardiac death (IDDM vs. NIDDM vs. non-diabetics)
**Figure S4.** Diabetes-related ten-year cumulative incidence of TLR (IDDM vs. NIDDM vs. non-diabetics)
**Figure S5.** Diabetes-related ten-year cumulative incidence of MI (IDDM vs. NIDDM vs. non-diabetics) **Figure S6.** Three-year outcomes in time deciles based on time of first PCI of DES-ISR
**Figure S7.** Landmark analysis of cardiac death by diabetes
**Figure S8.** Landmark analysis of TLR by diabetes
Abbreviations: CD, cardiac death; DES, drug-eluting stent; DM, diabetes mellitus; IDDM, insulin dependent diabetes mellitus; ISR, in-stent restenosis; MI, myocardial infarction; NIDDM, non-insulin dependent diabetes mellitus; PCI, percutaneous coronary intervention; TLR, target lesion revascularization

**Table S1.** Clinical baseline characteristics (IDDM vs. NIDDM vs. non-diabetics)

|  | **Patients with IDDM (n=471)** | **Patients with NIDDM**  **(n=771)** | **Non-diabetics (n=2,269)** | **P value** |
| --- | --- | --- | --- | --- |
| Age (in years)  Male sex  BMI (in kg/m^2^) | 69.3 ± 9.9  73.9 (348)  29.6 ± 5.1  (n=467) | 69.9 ± 9.6  77.6 (598)  28.1 ± 4.5  (n=766) | 69.0 ± 10.9  79.4 (1,802)  26.9 ± 4.2  (n=2,198) | 0.154  0.026  <0.001 |
| Arterial hypertension | 98.3 (463) | 97.7 (453) | 90.4 (2,052) | <0.001 |
| Hypercholesterolemia | 77.1 (363) | 75.9 (585) | 72.2 (1,639) | 0.028 |
| Current smoker | 15.1 (71) | 15.4 (119) | 14.5 (330) | 0.822 |
| CAD   - 1-vessel CAD - 2-vessel CAD - 3-vessel CAD | 6.2 (29)  15.9 (75)  77.9 (367) | 6.9 (53)  18.7 (144)  74.4 (574) | 12.4 (282)  21.5 (488)  66.1 (1,499) | <0.001 |
| Multivessel CAD  Prior MI  Prior CABG  Ejection fraction (in %) | 93.8 (442)  41.4 (195)  17.6 (83)  51.0 ± 11.5 | 93.1 (718)  44.9 (346)  14.1 (109)  51.6 ± 11.5 | 87.6 (1,987)  45.9 (1,042)  13.0 (296)  52.8 ± 10.9 | <0.001  0.198  0.032  0.001 |
| Clinical presentation   - Stable angina - Unstable angina - NSTEMI - STEMI   Creatinine ad admission (in mg/dl)  CRP ad admission (in mg/l)  ASA at discharge  P2Y12 inhibitor at discharge  Oral anticoagulation at discharge  Statin at discharge | 66.7 (314)  11.7 (55)  18.9 (89)  2.8 (13)  1.12 ± 0.44  6.0 ± 20.6  972 (458)  96.8 (456)  12.7 (60)  91.3 (430) | 71.7 (553)  12.2 (94)  13.7 (106)  2.3 (18)  1.10 ± 0.36  5.1 ± 14.3  95.8 (739)  94.4 (728)  14.1 (109)  90.0 (649) | 71.2 (1,615)  15.8 (358)  10.2 (232)  2.8 (64)  1.07 ± 0.45  4.9 ± 14.1  97.0 (2,200)  94.6 (2,147)  14.3 (324)  91.8 (2,082) | <0.001  0.029  0.362  0.264  0.119  0.679  0.331 |

Data shown as percentage (number) or means ± standard deviation
Abbreviations: ASA, acetylsalicylic acid; BMI, body mass index; CABG, coronary artery bypass graft; CAD, coronary artery disease; CRP, C-reactive protein; IDDM, insulin dependent diabetes mellitus; MI, myocardial infarction; NIDDM, non-insulin dependent diabetes mellitus; NSTEMI, non-ST-elevation myocardial infarction; STEMI, ST-elevation myocardial infarction

**Table S2.** Procedural characteristics (IDDM vs. NIDDM vs. non-diabetics)

|  | **Lesions in patients with IDDM (n=825)** | **Lesions in patients**  **with NIDDM (n=1,249)** | **Lesions in non-diabetics (n=3,423)** | **P value** |
| --- | --- | --- | --- | --- |
| Type of intervention   - Balloon angioplasty - DES implantation   Drug-coated balloon  Cutting balloon  Bifurcation lesion  Ostial lesion | 51.2 (422)  48.8 (403)  24.5 (202)  2.4 (20)  34.1 (281) (n=823)  32.9 (271) (n=824) | 47.3 (591)  52.7 (658)  19.8 (247)  2.8 (35)  34.2 (427)  31.5 (393) | 45.3 (1,550)  54.7 (1,873)  15.6 (534)  2.6 (88)  32.5 (1,105) (n=3,395)  30.0 (1,019) (n=3,398) | 0.009  <0.001  0.855  0.466  0.224 |
| Max. balloon diameter (mm)  Max. balloon pressure (atm)  Max. stent diameter (mm)  Total stented length (mm)  TIMI grade flow pre PCI   - 0 - 1 - 2 - 3 | 3.30 ± 0.63 (n=812)  15.8 ± 4.0 (n=810)  3.26 ± 0.56 (n=405)  24.6 ± 13.3 (n=405)  6.7 (55)  1.9 (16)  7.8 (64)  83.6 (690) | 3.0 (3.0; 3.5) (n=1,228)  16.0 ± 4.3 (n=1,230)  3.23 ± 0.54 (n=667)  25.5 ± 13.8 (n=670)  6.9 (86)  1.9 (24)  7.0 (87)  84.2 (1,051) | 3.33 ± 0.61 (n=3,305)  15.8 ± 4.1 (n=3,284)  3.28 ± 0.52 (n=1,861)  25.6 ± 13.2 (n=1,861)  8.2 (280)  1.9 (66)  6.3 (215)  83.5 (2,847) | 0.329  0.336  0.334  0.571  0.453 |
| TIMI grade flow post PCI   - 0 - 1 - 2 - 3 | 1.6 (13)  0.4 (3)  1.7 (14)  96.4 (795) | 1.4 (18)  0.1 (1)  2.2 (27)  96.3 (1,202) | 2.0 (68)  0.4 (14)  2.0 (69)  95.6 (3,254) | 0.486 |

Data shown as percentage (number) or means ± standard deviation
Abbreviations: DES, drug-eluting stent; IDDM, insulin dependent diabetes mellitus; NIDDM, non-insulin dependent diabetes mellitus; PCI, percutaneous coronary intervention; TIMI, thrombolysis in myocardial infarction

**Table S3.** Three-year outcomes in time deciles

|  | **P_int_ value (DM + PCI time)** | **P_int_ value (PCI type + PCI time)** | **P value (DM)** |
| --- | --- | --- | --- |
| Cardiac Death  TLR | 0.309  0.263 | 0.114  0.058 | <0.001  <0.001 |

PCI type: DES vs. DCB vs. POBA. PCI time: time of first PCI of DES-ISR
Abbreviations: DCB, drug-coated balloon; DES, drug-eluting stent; DM, diabetes mellitus; ISR, in-stent restenosis; PCI, percutaneous coronary intervention; POBA, plain old balloon angioplasty; TLR, target lesion revascularization

**Fig. S1**

The total cohort was divided into three equal groups: patients treated “early”, patients treated “recently” and patients treated in between (“middle”) for first DES-ISR. **a** represents the rate of DES implantation for the treatment of DES-ISR, **b** the rate of DCB use for the treatment of DES-ISR.

Abbreviations: DCB, drug-coated balloon; DES, drug-eluting stent; ISR, in-stent restenosis

**Fig. S2**

**
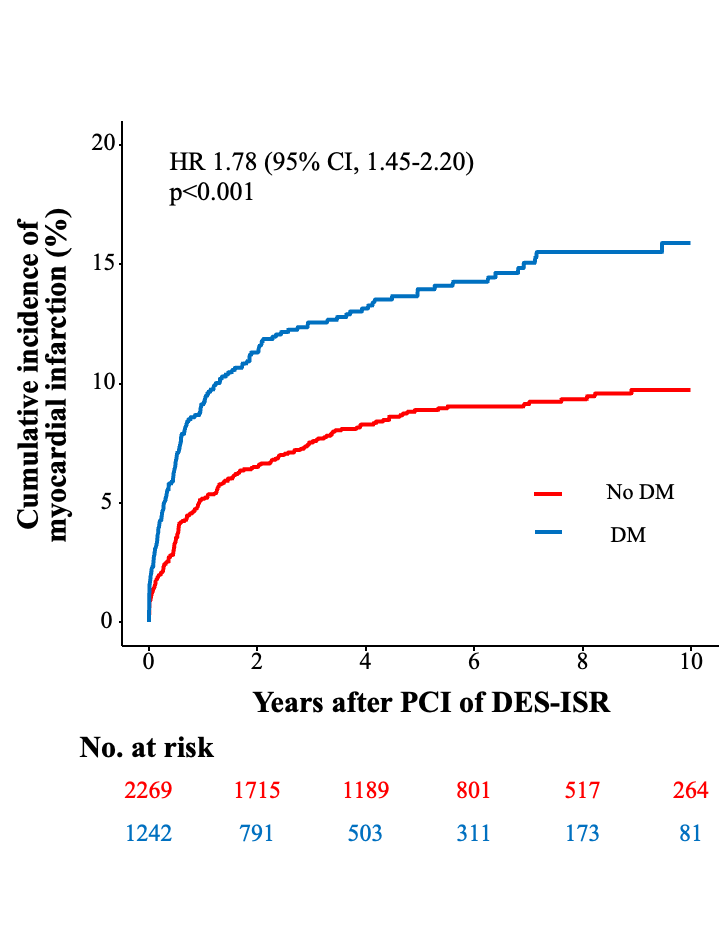
**

**Fig. S3**

**
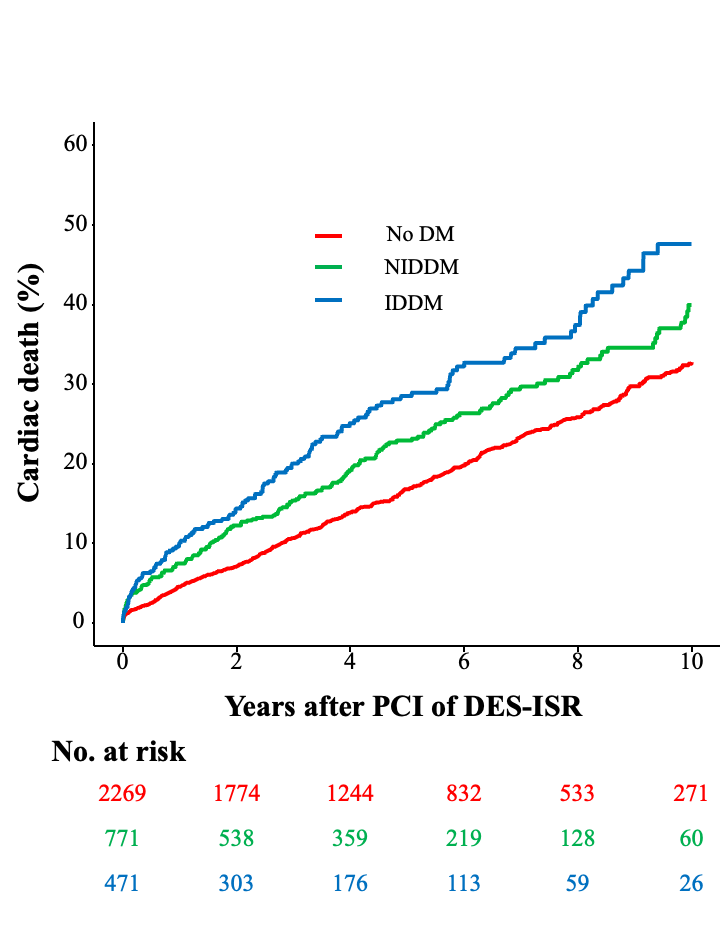
**

**Fig. S4**

**
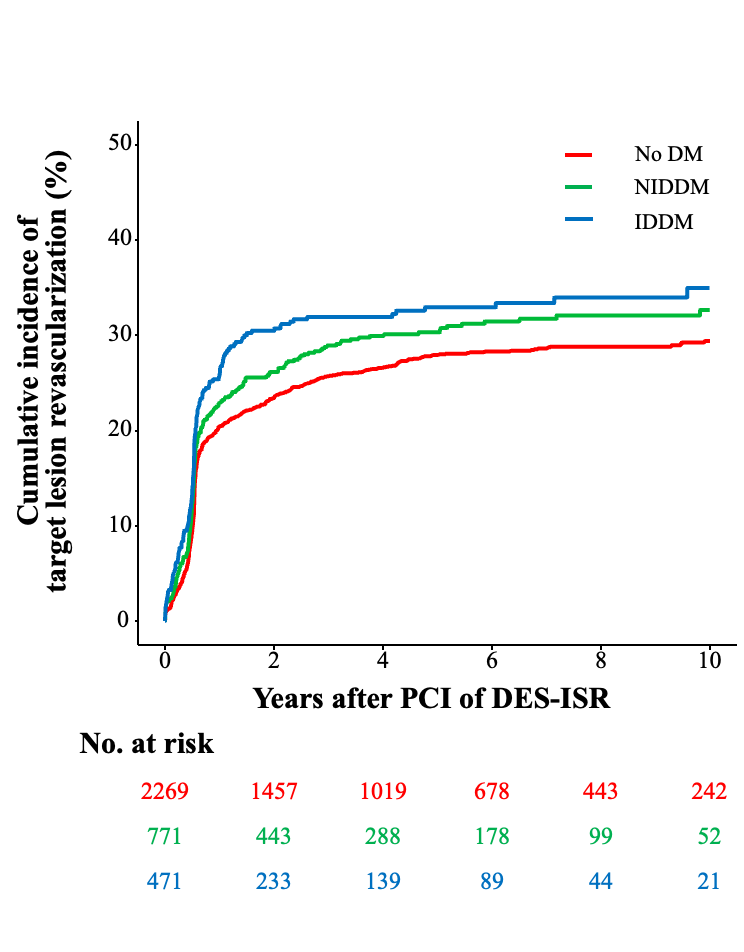
**

**Fig. S5**

**
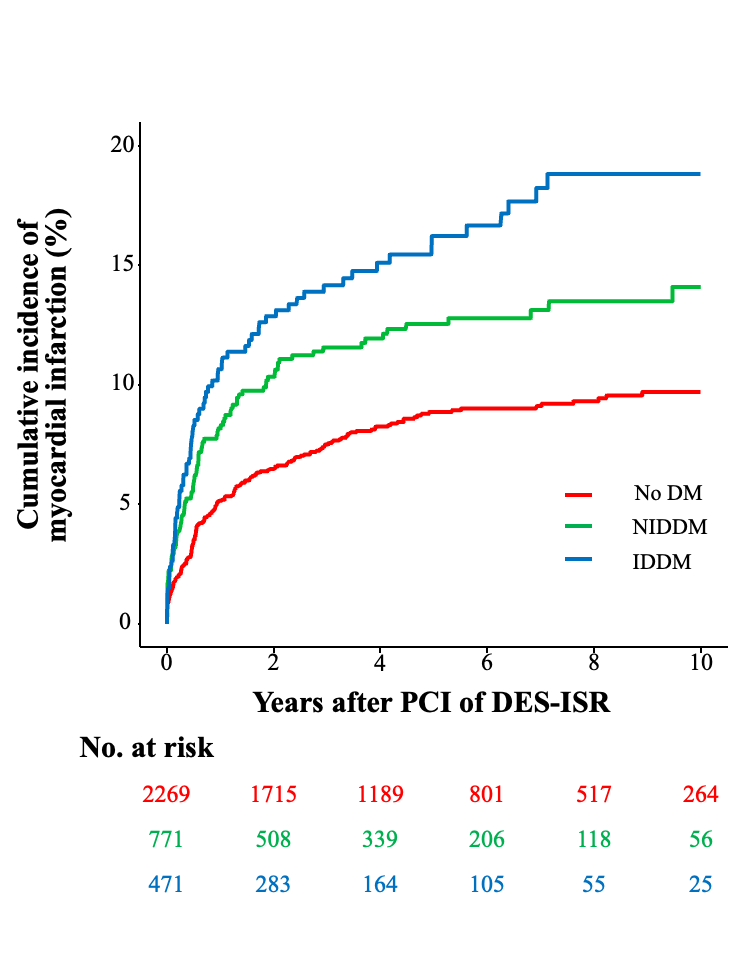
**

**Fig. S6**


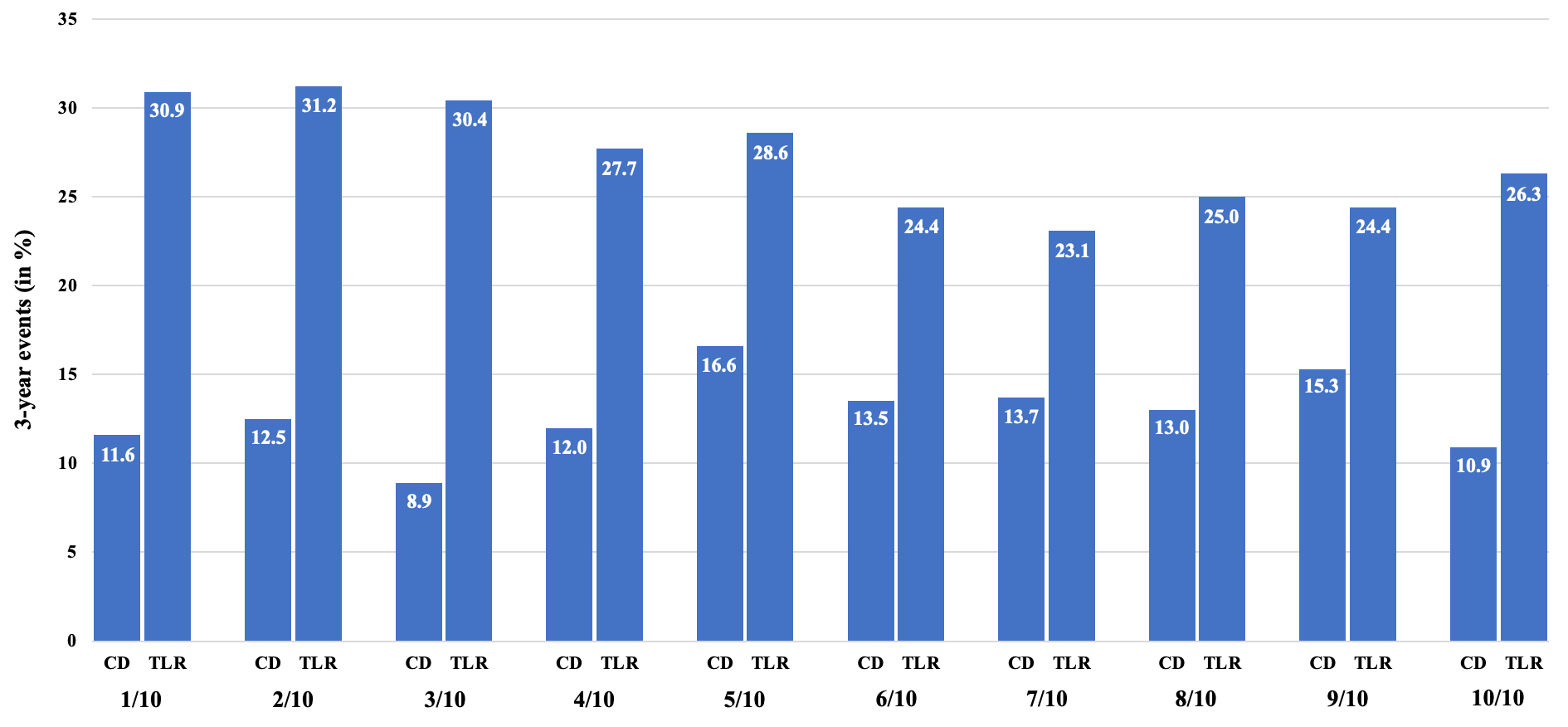


**Fig. S7**

**
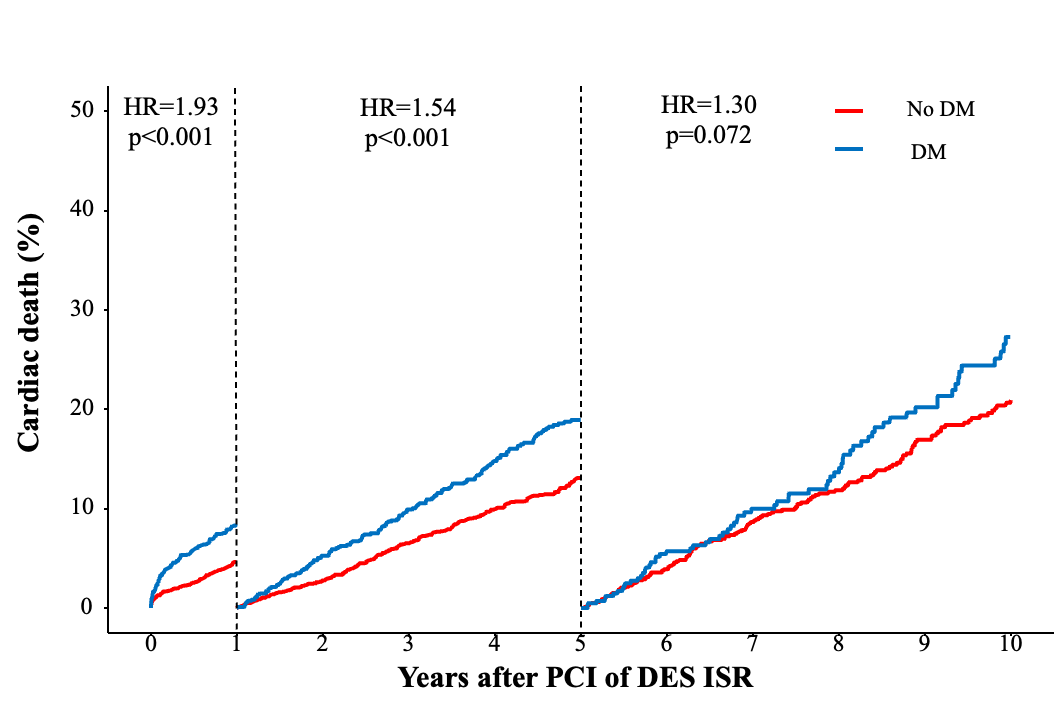
**

**Fig. S8**


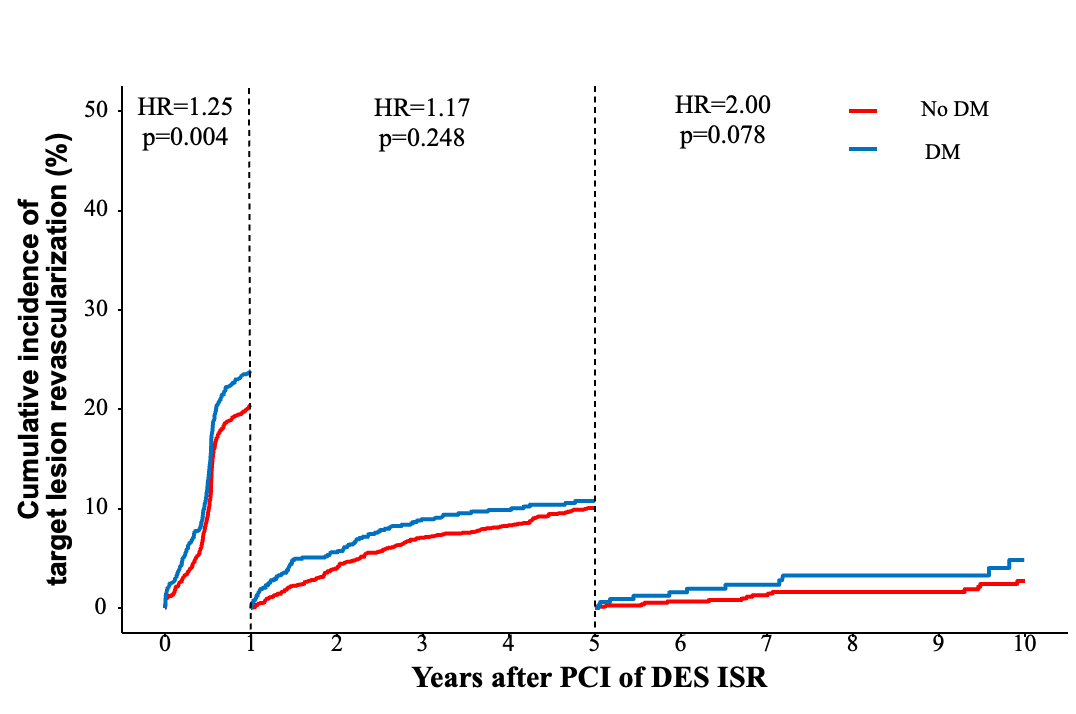

Supplement: Supplementary file 1 — (DOCX 557 KB) [file 392_2025_2782_MOESM1_ESM.docx]
